# Carvacrol Coadministration Ameliorates Lambda‐Cyhalothrin‐Induced Peripheral Neuropathy in Rats: Behavioral and Molecular Evidence

**DOI:** 10.1002/jbt.70400

**Published:** 2025-07-15

**Authors:** Özge Kandemir, Mustafa İleritürk, Cihan Gür, Nurhan Akaras, Hasan Şimşek, Selçuk Yılmaz, Fatih Mehmet Kandemir

**Affiliations:** ^1^ Department of Food Processing, Aksaray Technical Sciences Vocational School Aksaray University Aksaray Turkey; ^2^ Department of Animal Science, Horasan Vocational College Atatürk University Erzurum Turkey; ^3^ Department of Medical Laboratory Techniques, Vocational School of Health Services Atatürk University Erzurum Turkey; ^4^ Department of Histology and Embryology, Faculty of Medicine Aksaray University Aksaray Turkey; ^5^ Department of Physiology, Faculty of Medicine Aksaray University Aksaray Turkey; ^6^ Department of Orthopedics and Traumatology Antalya City Hospital Antalya Turkey; ^7^ Department of Medical Biochemistry Faculty of Medicine, Aksaray University Aksaray Turkey

**Keywords:** carvacrol, cyhalothrin, oxidative stress, peripheral neuropathy, sciatic nerve

## Abstract

This study aimed to investigate the possible neuroprotective effects of Carvacrol (CRV) against Lambda‐cyhalothrin (CYH)‐induced peripheral neuropathy. Thirty‐five rats were divided into five groups: Control, CRV, CYH, CYH+CRV25, and CYH+CRV50. CRV 25 or 50 mg/kg and CYH 6.23 mg/kg were administered orally for 21 days. The effects of these treatments were evaluated by hot plate and rotarod tests, followed by molecular, biochemical, histopathological, and immunohistochemical analyses of sciatic nerve tissues. CYH administration significantly impaired both sensory and motor functions. CRV doses (25 mg/kg and 50 mg/kg) administered with CYH significantly improved these impairments (*p* < 0.001). Additionally, CYH increased MDA levels and decreased antioxidants, while CRV treatment reversed these effects. CRV also suppressed inflammation (*p* < 0.01), apoptosis (*p* < 0.001), and endoplasmic reticulum stress (*p* < 0.001), with the 50 mg/kg dose being more effective. Morphological and immunohistochemical analyses showed that CRV treatment partially repaired CYH‐induced nerve damage, with both doses reducing 8‐OHdG and beclin‐1 immunoreactions. The data revealed that CYH induced inflammation, oxidative stress, ER stress, and apoptosis in sciatic tissue, while CRV exhibited antioxidant, anti‐inflammatory, and antiapoptotic effects, reducing the damage and suggesting its potential as a supportive treatment for CYH‐induced sciatic damage.

## Summary

- Lambda‐Cyhalothrin induces neurotoxicity.
- Carvacrol ameliorates Lambda‐Cyhalothrin‐induced oxidative stress injury.
- Carvacrol ameliorates Lambda‐Cyhalothrin‐induced neuroinflammation.
- Carvacrol ameliorates Lambda‐Cyhalothrin‐induced apoptotic injury.
- Carvacrol ameliorates Lambda‐Cyhalothrin‐induced endoplasmic reticulum stress.
- Carvacrol improves motor functions impaired by Lambda‐Cyhalothrin.

## Graphical abstract

This study investigates the peripheral neuropathy induced by the pesticide Lambda‐cyhalothrin (CYH) and how carvacrol (CRV) treatment reduces these effects. CYH causes neurological disorders, oxidative stress, and inflammation, while CRV reverses these harmful effects with its antioxidant, anti‐inflammatory, and antiapoptotic properties.

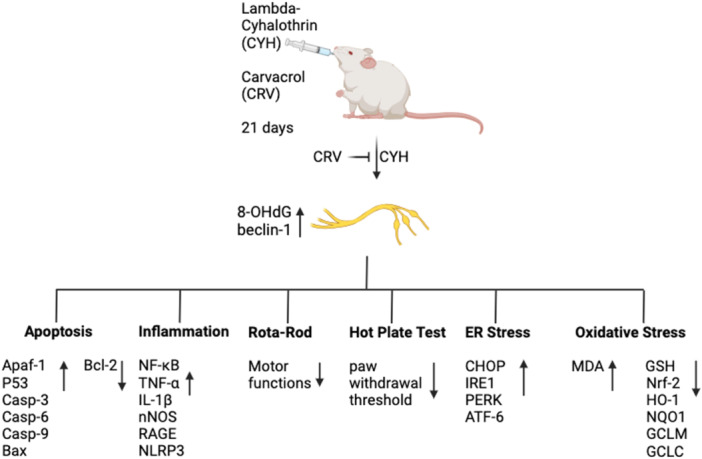

**Abbreviations**
Apaf‐1: apoptotic protease activating factor 1
ATF‐6: activating transcription factor 6
Bax: Bcl‐2‐associated X protein
Bcl‐2: B‐cell lymphoma 2
Casp‐3: cysteine‐aspartic acid protease‐3
Casp‐6: cysteine‐aspartic acid protease‐6
Casp‐9: cysteine‐aspartic acid protease‐9
CAT: catalase
CHOP: C/EBP homologous protein
COX‐2: cyclooxygenase‐2
CRV: carvacrol
CYH: lambda‐cyhalothrin
ERS: endoplasmic reticulum stress
GCLC: glutamate cysteine ligase catalytic subunit
GCLM: glutamate cysteine ligase modifier subunit
GPx: glutathione peroxidase
GSH: glutathione
HO‐1: heme oxygenase‐1
IL‐1β: interloukin‐1β
IRE1: inositol‐requiring enzyme 1
Keap‐1: kelch‐like ECH‐associated protein 1
MDA: malondialdehyde
NF‐κB: nuclear factor kappa‐B
NLRP3: NLR family pyrin domain containing 3
nNOS: neuronal nitric oxide synthase
NOS: nitric oxide synthase
NQO1: NAD(P)H quinone dehydrogenase 1
Nrf2: nuclear factor erythroid 2–related factor 2
P53: tumor suppressor protein
PERK: protein kinase RNA‐like ER kinase
PGE‐2: prostaglandin E2
PVDF: polyvinylidene fluoride
RAGE: receptor for advanced glycation end products
RIPA: radioimmunoprecipitation assay buffer
ROS: reactive oxygen species
SDS/PAGE: sodium dodecylsulfate polyacrylamide gel electrophoresis
SOD: superoxide dismutase
TNF‐α: tumor necrosis factor alpha

## 1 Introduction

Pyrethroids are among the most widely used insecticides in the world [1]. Pyrethroids are synthetic chemicals developed based on pyrethrin structures and were developed from flower extracts of *Chrysanthemum cinerariaefolium* in the 1970s and began to be used to control insect pests on animals, agriculture, and public health [2]. Pyrethrins, which are divided into 2 types, cause paralysis, hyperexcitability, and tremors at acute toxic doses, while type II pyrethrins cause hypersensitivity, choreoathetosis, and salivation [3]. Lambda‐cyhalothrin (CYH or λ‐Cyhalothrin: α‐cyano‐3‐phenoxybenzyl‐3‐(2‐chloro‐3,3,3‐trifluoro‐propenyl)‐2,2‐dimethyl cyclopropane carboxylate) is a type II pyrethroid. It affects the eggs and larvae of flies, mosquitoes, ants, and cockroaches, and is used both in rural areas and indoors [4]. Although it is mentioned that its nontarget organism toxicity is low, human exposure is increasing due to widespread use [5]. This active ingredient is becoming a more widely used pesticide than organochlorines and organophosphates, so demand for CYH is expected to exceed $3.8 billion by 2027 [6]. Pyrethrins, including CYHs, cause continuous neuronal discharge by stimulating sodium channels in nerve membranes. In addition, their lipophilic structure facilitates absorption and accumulation in fat‐rich tissues, allowing direct access to the nervous system via sensory organs of peripheral nerves [1]. Moreover, CYHs tend to accumulate in biological membranes, causing oxidative stress and inflammation [7].

Compounds obtained from natural plant sources have begun to become the focus of research to be used in the treatment of many pathological conditions because they have different biological and pharmacological properties [8]. Carvacrol (CRV) is a monoterpene compound and is found in thyme, black pepper, wild bergamot, and marjoram essential oil. CRV, which is generally used as a sweetener and food additive, is known to have anti‐inflammatory, antioxidant, antitumor, antiapoptotic, and antimicrobial effects [9]. The presence of a hydroxyl group (OH) covalently bonded to the aromatic ring in its structure is the most important factor in CRV's antioxidant activity [10]. Inhibition of prostaglandin synthesis, suppression of COX‐2, and reduction of IL‐1β and PGE2 levels are the results of CRV's anti‐inflammatory effect [11, 12].

In the study, the therapeutic effects of CRV in the CYH‐induced peripheral neuropathy model were investigated comprehensively through behavioral observations and molecular, biochemical, and histopathological analyses.

## 2 Materials and Methods

### 2.1 Chemicals

CYH (CAS No. 91465‐08‐6) and CRV (CAS No. 499‐75‐2) used in the study were supplied by Sigma Chemical Company (St. Louis, MO, USA). Other chemicals used in the analyses were of analytical grade and were purchased from the same company.

### 2.2 Animals and Ethics Approval

Thirty‐five male *Sprague‐Dawley* rats, 10‐12 weeks old and weighing 220–250 g, were obtained from Atatürk University Medical Experimental Application and Research Center. The animals were kept at 24 ± 1°C, 45 ± 5% humidity, and a 12‐h light/dark cycle with ad libitum food and water.

### 2.3 Experimental Protocols

Rats were divided into 5 groups, each consisting of 7 rats. The study conducted by İleritürk and Kandemir [13] was used to determine the doses of CYH and CRV. A total of five different groups, each consisting of seven animals, were randomly selected for the experiment. The groups were designed as follows:

Control group: Oral physiological saline was administered for 21 days.
CRV group: Oral carvacrol (50 mg/kg) was administered for 21 days.
CYH group: Oral λ‐Cyhalothrin (6.23 mg/kg) was administered for 21 days.
CYH + CRV25 group: Oral carvacrol (25 mg/kg) was administered for 21 days, followed by oral λ‐Cyhalothrin (6.23 mg/kg) 30 min later.
CYH+CRV50 group: λ‐Cyhalothrin (6.23 mg/kg) was administered orally 30 min after oral carvacrol (50 mg/kg) administration for 21 days.

The effects of CYH and CRV on sensory nerve functions were evaluated on day 22 with hot plate and rotarod tests according to the test protocols detailed below. The tests were performed by a researcher unaware of the treatment.

### 2.4 Hot Plate Test

The purpose of this test was to measure the heat sensitivity of rats. The test was performed according to the method used by Semiş et al. [14]. For this purpose, a plate set at 52°C was used. The time between the moment the rats touched the plate with their paws and the moment they first removed their paws from the plate was determined.

### 2.5 Rotarod Test

The Rotarod test was performed using the method of Semis et al. [14]. For this purpose, a Rotarod apparatus (Ugo Basile, Varese, Italy) with a diameter of 6 cm and a height of 25 cm was used. Before the measurements, the animals were allowed to adapt to the device for 1 week. Measurements were made after the end of the drug administrations. The rotation speed of the rod was 10 rpm. The duration of the animals’ stay on the rod was measured.

### 2.6 Tissue Collection

After the behavioral tests, the rats were decapitated with light sevoflurane (Sevorane 100% liquid; Abbott Laboratories) anesthesia, and the sciatic nerve tissues were removed. Some of the sciatic nerve tissues, which were cleaned of membranes and clots, were kept at −80°C for biochemical and molecular analyses, while others were stored in 10% buffered formaldehyde for histopathological analyses.

### 2.7 MDA and GSH Analysis in Sciatic Nerve Tissue

Sciatic nerve tissue was ground with liquid nitrogen (Tissue Lyser II, Qiagen) and homogenized in 1.15% potassium chloride buffer at a ratio of 1:10 (w/v). A portion of the homogenate was centrifuged at 10,000 rpm for 20 min at 4°C and the supernatant obtained was used to measure GSH levels. The remaining homogenate was centrifuged at 3500 rpm for 15 min and used for MDA analysis. The methods of Placer et al. [15] were used to analyze the levels of MDA, the end product of lipid peroxidation, and Sedlak and Lindsay [16] were used to measure the levels of GSH.

### 2.8 RT‐PCR Analyses on Sciatic Nerve Tissue

Sciatic nerve tissues taken from rats were treated with QIAzol Lysis Reagent (79306; Qiagen) to isolate total RNAs. All procedures were performed according to the manufacturer's instructions. The concentrations of the obtained total RNAs were measured on the NanoDrop (BioTek Epoch) device. In the next step, cDNA synthesis was performed from total RNAs using the iScript cDNA Synthesis Kit (Bio‐Rad) according to the manufacturer's instructions, and cDNAs were reacted with iTaq Universal SYBR Green Supermix (BIO‐RAD) and primers (Table 1) on the Rotor‐Gene Q (Qiagen) device. After the reactions were completed, genes were normalized to β‐actin by the 2^−ΔΔCT^ method [17].

**Table 1:** Primer sequences.

Gene	Sequences (5′‐3′)	Length (bp)	Accession No
Nrf2	F: TTTGTAGATGACCATGAGTCGC R: TCCTGCCAAACTTGCTCCAT	161	NM_031789.2
HO‐1	F: ATGTCCCAGGATTTGTCCGA R: ATGGTACAAGGAGGCCATCA	144	NM_012580.2
NQO1	F: CTGGCCAATTCAGAGTGGCA R: GATCTGGTTGTCGGCTGGAA	304	NM_017000.3
GCLM	F: ACCAGTGGGCACAGGTAAAA R: CCACTCCTGGGCTTCAATGT	177	NM_017305.2
GCLC	F: TCCACTGTCCAAGGTTGACG R: GTGTCCACGTCGACTTCCAT	270	NM_012815.2
NF‐κB	F: AGTCCCGCCCCTTCTAAAAC R: CAATGGCCTCTGTGTAGCCC	106	NM_001276711.1
TNF‐α	F: CTCGAGTGACAAGCCCGTAG R: ATCTGCTGGTACCACCAGTT	139	NM_012675.3
IL‐1β	F: ATGGCAACTGTCCCTGAACT R: AGTGACACTGCCTTCCTGAA	197	NM_031512.2
nNOS	F: TGGAGACATCATTCTCGCAG R: GATGTGTAGTGAAGCCCTCA	140	NM_052799.2
RAGE	F: CTGAGGTAGGGCATGAGGATG R: TTCATCACCGGTTTCTGTGACC	113	NM_053336.2
NLRP3	F: TCCTGCAGAGCCTACAGTTG R: GGCTTGCAGCACTGAAGAAC	185	NM_001191642.1
Bcl‐2	F: GACTTTGCAGAGATGTCCAG R: TCAGGTACTCAGTCATCCAC	214	NM_016993.2
Bax	F: TTTCATCCAGGATCGAGCAG R: AATCATCCTCTGCAGCTCCA	154	NM_017059.2
P53	F: GCGCTTCGAGATGTTCCGA R: AGACTGGCCCTTCTTGGTCT	121	NM_030989.3
Apaf‐1	F: ACCTGAGGTGTCAGGACC R: CCGTCGAGCATGAGCCAA	192	NM_023979.2
Caspase‐3	F: ACTGGAATGTCAGCTCGCAA R: GCAGTAGTCGCCTCTGAAGA	270	NM_012922.2
Caspase‐6	F: AGACCTTGACTGGCTTGTTCA R: TCTGTCTGATGATCCACCACG	139	NM_001271984.1
Caspase‐9	F: ACGTGAACTTCTGCCCTTCC R: GGTCGTTCTTCACCTCCACC	117	NM_031632.2
IRE1	F: GCAGTTCCAGTACATTGCCATTG R: CAGGTCTCTGTGAACAATGTTGA	163	NM_001191926.1
PERK	F: GATGCCGAGAATCATGGGAA R: AGATTCGAGAAGGGACTCCA	198	NM_031599.2
ATF‐6	F: TCAACTCAGCACGTTCCTGA R: GACCAGTGACAGGCTTCTCT	130	NM_001107196.1
CHOP	F: GAAGCCTGGTATGAGGATCT R: GAACTCTGACTGGAATCTGG	209	NM_001109986.1
β‐Actin	F: CAGCCTTCCTTCTTGGGTATG R: AGCTCAGTAACAGTCCGCCT	360	NM_031144.3

### 2.9 Western Blot Analysis

Western blot analysis was performed following protocols similar to those used in previous studies [13]. The tissues were pulverized using liquid nitrogen and homogenized in RIPA buffer containing a protease inhibitor cocktail and PMSF. Homogenates were centrifuged at 16,000 × *g* for 20 min, and the total protein content in the supernatant was determined using the Pierce™ BCA Protein Assay Kit (Thermo Fisher Scientific, Waltham, MA, USA). A total of 50 µg of protein was loaded into each well and separated by 10% SDS‐PAGE. The separated proteins were then transferred onto PVDF (polyvinylidene fluoride) membranes and blocked for 1.5 h with 5% BSA dissolved in PBS‐T (phosphate‐buffered saline containing 0.1% Tween 20). Following blocking, the membranes were incubated overnight at 4°C with primary antibodies at a 1:500 dilution (Nrf2, sc‐365949; HO‐1, sc‐136960; Caspase‐3, sc‐7272; Bax, sc‐7480; Bcl‐2, sc‐7382; β‐Actin, sc‐8432). The membranes were then washed with PBS‐T and treated with HRP‐conjugated secondary antibodies (1:2000 dilution) for 1.5 h. After the final wash with PBS‐T, the protein bands were visualized using Bio‐Rad Clarity Max ECL substrate (Bio‐Rad, Hercules, USA) and imaged with the Bio‐Rad Gel Doc XR+ Imaging System (Bio‐Rad, Hercules, USA). Densitometric analysis of the blots was conducted using the ImageLab software (Bio‐Rad, Hercules, USA). Each sample was analyzed with at least three replicates to ensure accuracy and reliability.

### 2.10 Morphological Evaluation of Sciatic Nerves

The rats were dissected under anesthesia 24 h after the last drug administration to obtain the sciatic nerve. The tissues were fixed in 10% formalin for 72 h. After the routine paraffin embedding procedure, 5 μm thick cross‐sections were taken from the paraffin‐embedded tissues. The hematoxylin‐eosin staining technique was used to see histopathological changes in the samples. Then, the stained sciatic nerves were examined under an optical microscope (Cx43, Olympus Inc., Tokyo, Japan) and photographed at ×200 magnification.

### 2.11 Immunohistochemical Analysis

After fixing the sciatic nerve tissue in formalin, a routine paraffin embedding procedure was applied. 3 μm thick sections were taken from the obtained paraffin blocks. The immunohistochemistry method was used to detect the expressions of some specific proteins secreted by the cells. The antigen retrieval process was applied to the tissues in citrate buffer solution at high temperatures. Then, endogenous peroxidase activity was prevented by waiting in hydrogen peroxide solution in the dark for 10 min. Primary antibodies (8‐OHdG (SC‐66036, Santa Cruz Biotechnology, 1:100 dilution), beclin‐1 (PA1‐16857, Invitrogen, 1:100 dilution) were dropped onto the slides placed in a humidified medium container and left overnight. After this process, secondary antibodies and streptavidin horseradish peroxidase were applied. After this stage, 3,3′‐diaminobenzidine tetrahydrochloride (DAB Substrate System (RTU), Thermo) solution was used to show the reactions. Then, the preparations were kept in hematoxylin dye for 5 min and passed through alcohol and xylene. The stained sections were examined using a binocular light microscope (Cx43, Olympus Inc., Tokyo, Japan) and photographed with a camera at x400 magnification. Grading according to immunohistochemical staining was done for each section. It was based on a score of 0–3. All samples were scored as follows: (0) absent, (1) minimal staining, (2) moderate staining, and (3) extensive staining.

### 2.12 Statistical Analysis

One‐way analysis of variance followed by Tukey's post‐hoc test was performed using GraphPad Prism 5.0. All values are expressed as mean ± standard deviation (SD), and *p* < 0.05 was considered significant.

## 3 Results

### 3.1 Evaluation of Behavioral Tests

#### 3.1.1 Hot Plate Test

**Figure 1:**
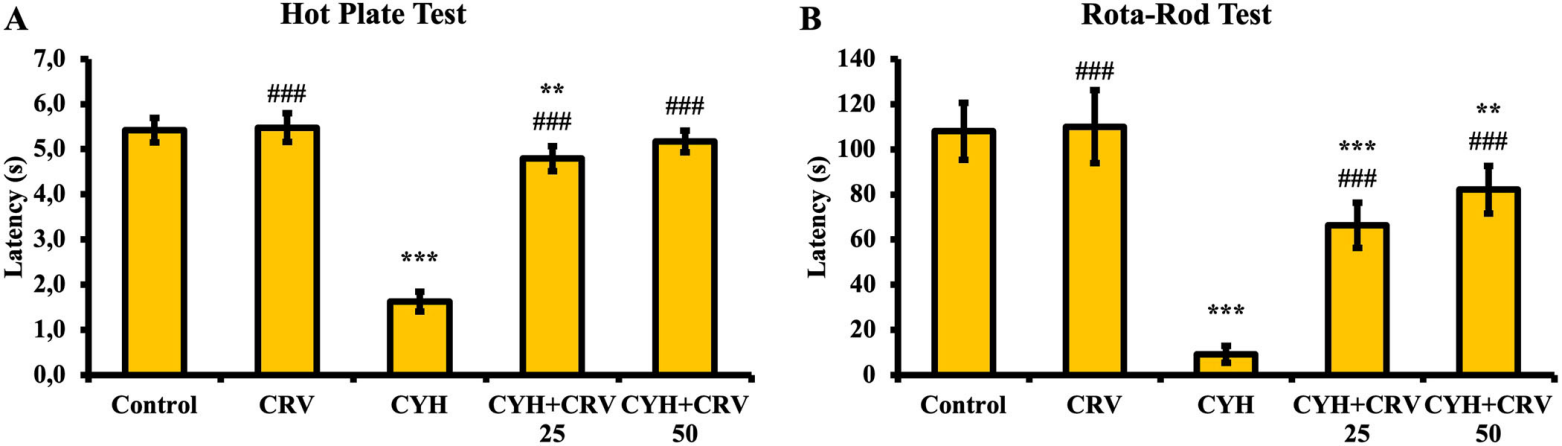
Hot plate test (A) and rotarod test (B) data after CYH and CRV administrations to rats. Data were given as mean ± SD. Control vs Others; ^⁎⁎⁎^ *p* < 0.001 and ^⁎⁎^ *p* < 0.01 CYH vs Others; ^###^ *p* < 0.001. CYH + CRV 25 vs CYH + CRV 50.

As a result of the hot plate test applied to rats (Figure 1A), it was determined that CYH application negatively affected sciatic nerve sensitivity and decreased paw withdrawal threshold compared to the control and CRV groups (*p* < 0.001). It was determined that CRV25 and CRV50 doses administered together with CYH were effective in increasing paw withdrawal threshold compared to the CYH group and reduced CYH‐induced sciatic nerve response loss (*p* < 0.001).

#### 3.1.2 Rotarod Test

The rotarod test was performed to evaluate the effects of CYH and CRV on motor functions in rats (Figure 1B). According to the findings, it was determined that CYH administration almost completely impaired motor functions compared to the control and CRV groups (*p* < 0.001), and both doses of CRV administered together with CYH were effective in improving impaired motor functions (*p* < 0.001).

### 3.2 Findings Regarding MDA and GSH Levels

**Figure 2:**
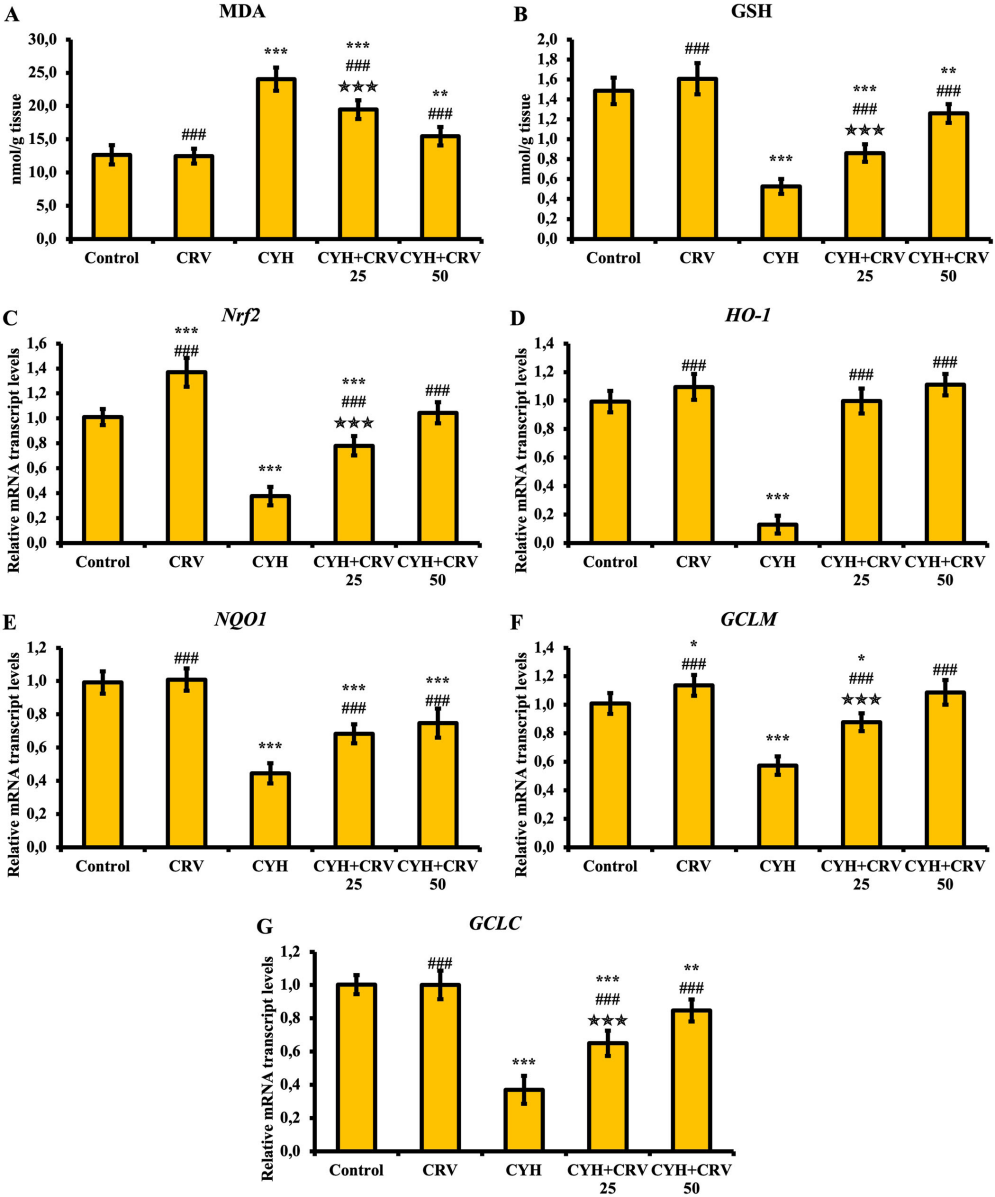
MDA (A) and GSH (B) levels and Nrf2 (C), HO‐1 (D), NQO1 (E), GCLM (F) and GCLC (G) mRNA transcript levels after CYH and CRV administration to rats. Data were given as mean ± SD. Control vs Others; ^⁎⁎⁎^ *p* < 0.001, ^⁎⁎^ *p* < 0.01, ^⁎^ *p* < 0.05. CYH vs Others; ^###^ *p* < 0.001. CYH + CRV 25 vs CYH + CRV 50; 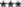 *p* < 0.05.

When the sciatic nerve tissue MDA (Figure 2A) and GSH (Figure 2B) levels were evaluated, it was determined that the levels of MDA, the end product of lipid peroxidation, increased in the sciatic nerve tissues of rats administered CYH (*p* < 0.001), whereas the levels of GSH, a nonenzymatic antioxidant, decreased (*p* < 0.001). It was determined that CRV administration together with CYH managed to reversely regulate these parameters in both doses, suppressing the sciatic nerve tissue MDA level and increasing the GSH level.

### 3.3 Findings on the Activities of Endogenous Antioxidants

The effects of CYH and CRV administrations on rat sciatic nerve tissue on endogenous antioxidant gene activation were evaluated by examining the expression levels of Nrf2, HO‐1, NQO1, GCLM, and GCLC (Figure 2). According to the findings, it was determined that CYH administration suppressed endogenous antioxidant gene activation and significantly reduced the expression levels of Nrf2 (C), HO‐1 (D), NQO1 (E), GCLM (F), and GCLC (G) (*p* < 0.001). In addition, it was determined that CRV administered together with CYH was effective at both doses and increased the expressions of these genes (*p* < 0.001).

### 3.4 Findings of Inflammatory Markers

**Figure 3:**
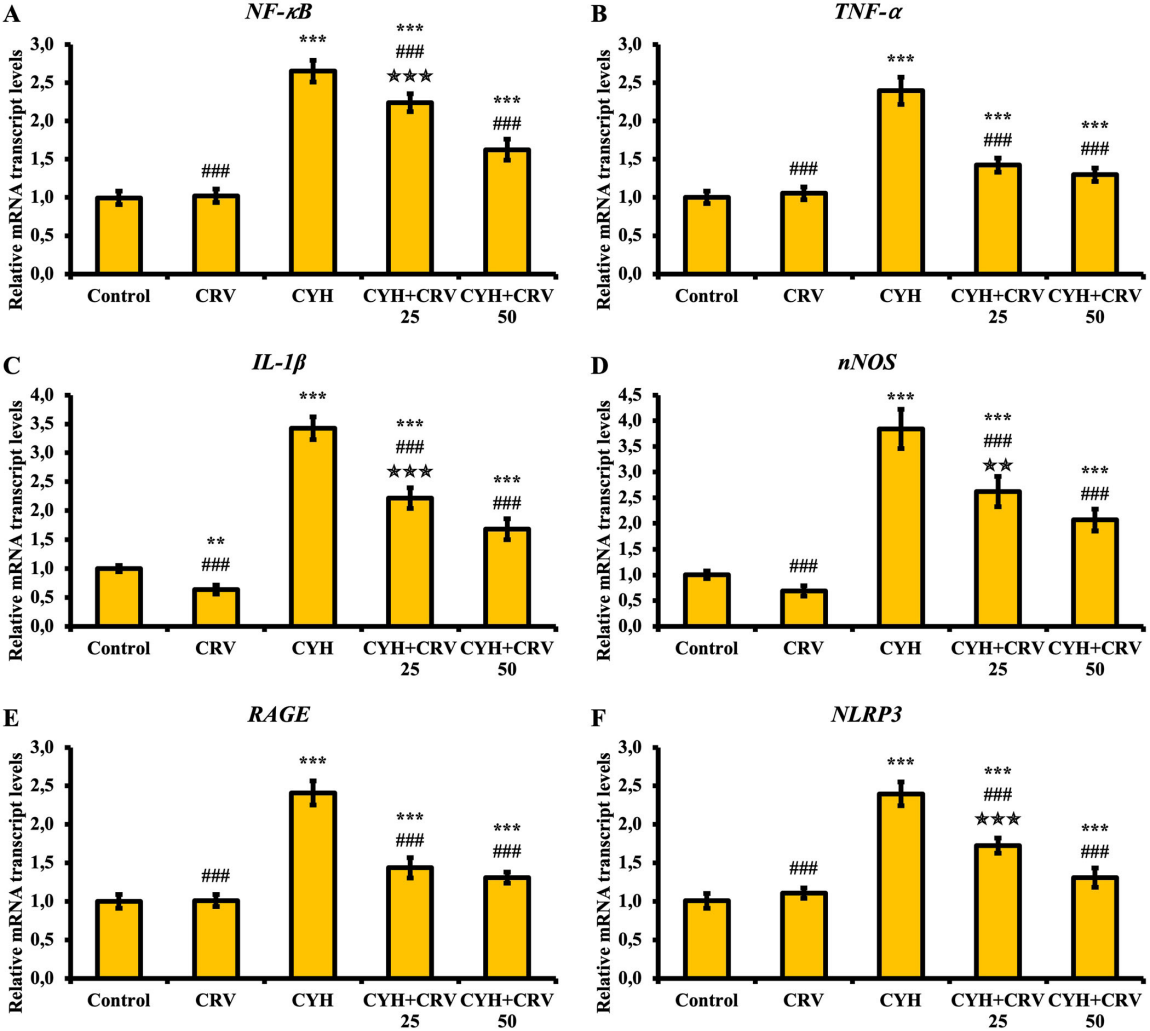
NF‐κB (A), TNF‐α (B), IL‐1β (C), nNOS (D), RAGE (E) and NLRP3 (F) mRNA transcript levels after CYH and CRV administration to rats. Data were given as mean ± SD. Control vs Others; ^⁎⁎⁎^ *p* < 0.001 and ^⁎⁎^ *p* < 0.01. CYH vs Others; ^###^ *p* < 0.001. CYH + CRV 25 vs CYH + CRV 50; 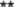 *p* < 0.01 and 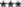 *p* < 0.05.

The effects of CYH and CRV administered to rats on sciatic nerve tissue inflammation were examined using NF‐κB, TNF‐α, IL‐1β, nNOS, RAGE, and NLRP3 markers (Figure 3). According to the findings, it was determined that CYH administration significantly increased NF‐κB (A), TNF‐α (B), IL‐1β (C), nNOS (D), RAGE (E) and NLRP3 (F) levels compared to the control and CRV groups and caused inflammation (*p* < 0.001), that CRV administration was effective at both doses and suppressed inflammation (*p* < 0.01), and that especially the CRV50 dose was more effective than the CRV25 dose (*p* < 0.05).

### 3.5 Findings of Apoptotic Markers

**Figure 4:**
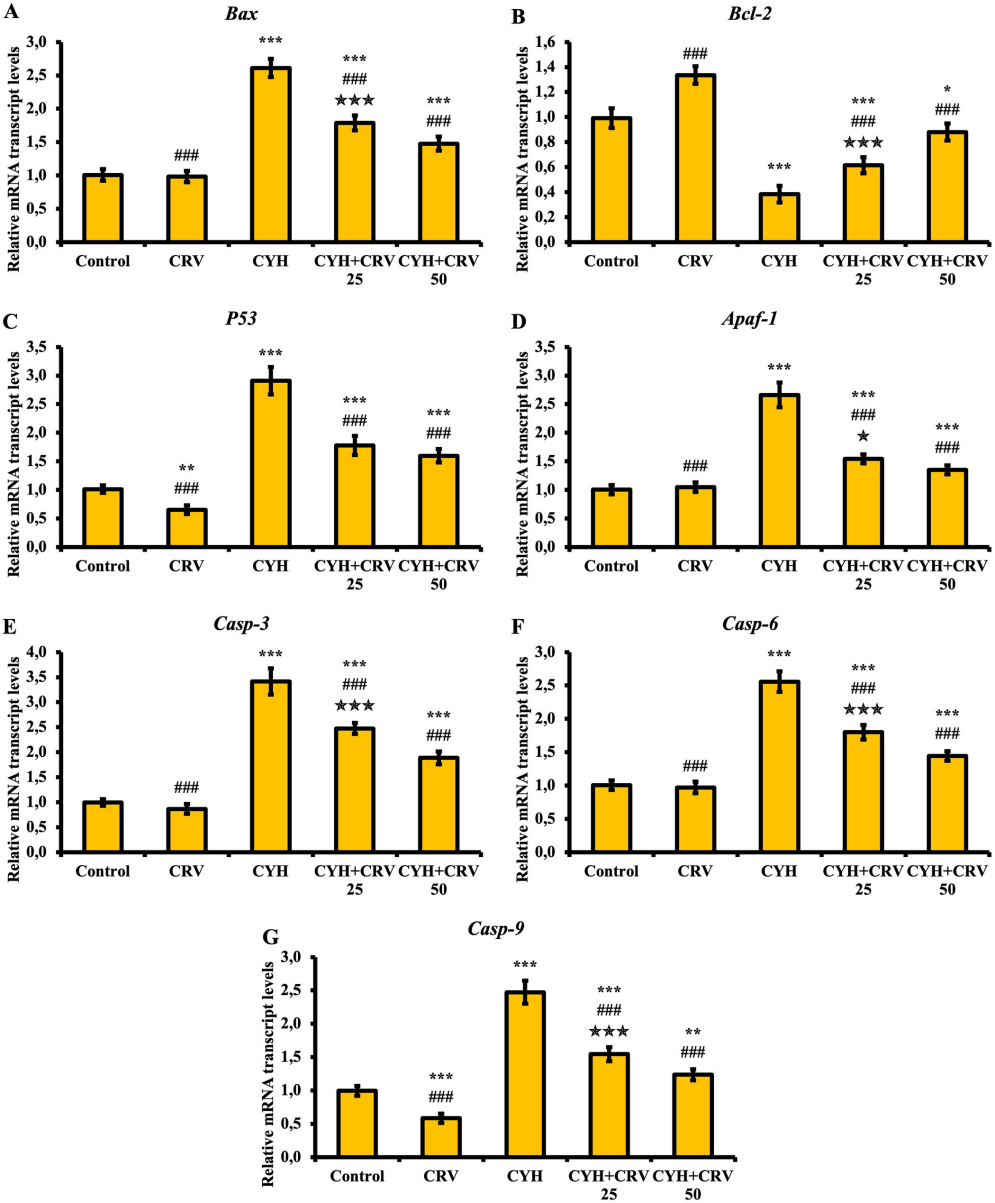
Bax (A), Bcl‐2 (B), P53 (C), Apaf‐1 (D), Casp‐3 (E), Casp‐6 (F) and Casp‐9 (G) mRNA transcript levels after CYH and CRV administration to rats. Data were given as mean ± SD. Control vs Others; ^⁎⁎⁎^ *p* < 0.001, ^⁎⁎^ *p* < 0.01, ^⁎^ *p* < 0.05. CYH vs Others; ^###^ *p* < 0.001. CYH + CRV 25 vs CYH + CRV 50; 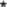 *p* < 0.001 and 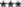 *p* < 0.05.

The effects of CYH and CRV administrations on sciatic nerve tissue apoptosis were examined with Bax, Bcl‐2, P53 Apaf‐1, Casp‐3, Casp‐6, and Casp‐9 markers (Figure 4). According to the obtained data, it was determined that CYH administration activated the apoptotic pathway in sciatic tissue compared to the control and CRV groups, increased the levels of Bax (A), P53 (C), Apaf‐1 (D), Casp‐3 (E), Casp‐6 (F) and Casp‐9 (G), and decreased the level of antiapoptotic marker Bcl‐2 (B), thus inducing apoptosis (*p* < 0.001). It was also detected that CRV administration together with CYH was successful in suppressing the apoptotic pathway at both doses, increased the level of Bcl‐2, and decreased the level of apoptotic markers Bax, P53, Apaf‐1, Casp‐3, Casp‐6, and Casp‐9 (*p* < 0.001).

### 3.6 Findings of Endoplasmic Reticulum Stress Markers

**Figure 5:**
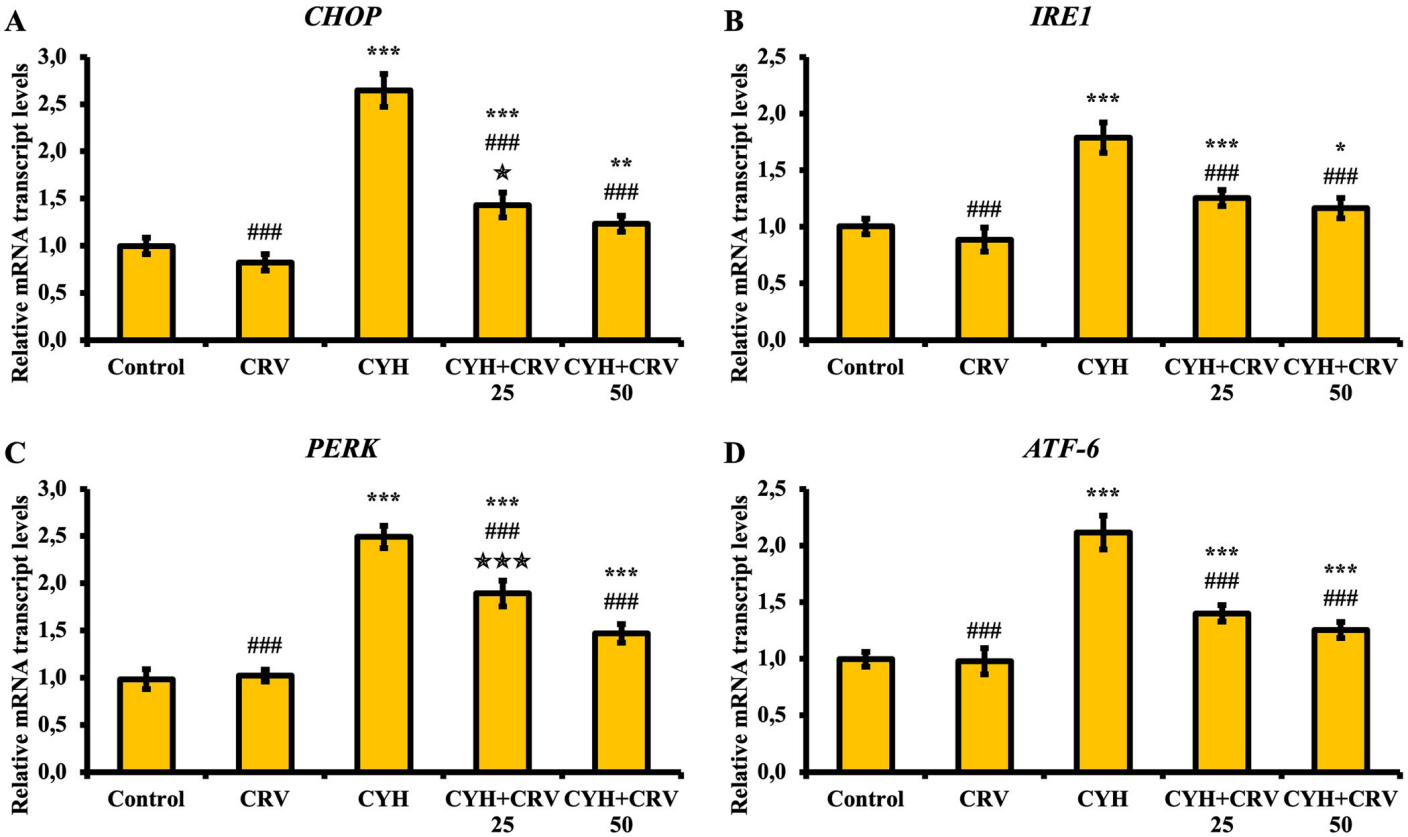
CHOP (A), IRE1 (B), PERK (C) and ATF‐6 (D) mRNA transcript levels after CYH and CRV administration to rats. Data were given as mean ± SD. Control vs Others; ^⁎⁎⁎^ *p* < 0.001, ^⁎⁎^ *p* < 0.01, ^⁎^ *p* < 0.05. CYH vs Others; ^###^ *p* < 0.001. CYH + CRV 25 vs CYH + CRV 50; 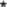 *p* < 0.001 and 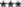 *p* < 0.05.

The expression levels of endoplasmic reticulum (ER) stress markers CHOP, IRE1, PERK, and ATF‐6 were measured in the sciatic nerve tissue and the results are given in Figure 5. Accordingly, it was found that CYH administration significantly increased the expression levels of CHOP (A), IRE1 (B), PERK (C), and ATF‐6 (D) compared to the control and CRV groups and triggered ER stress (*p* < 0.001), that CRV administration was effective in suppressing ER stress at both doses and reduced the expressions of CHOP, IRE1, PERK and ATF‐6 (*p* < 0.001), and that the CRV50 dose was more effective than the CRV25 dose in reducing ER stress (*p* < 0.05).

### 3.7 Findings of Protein Levels

**Figure 6:**
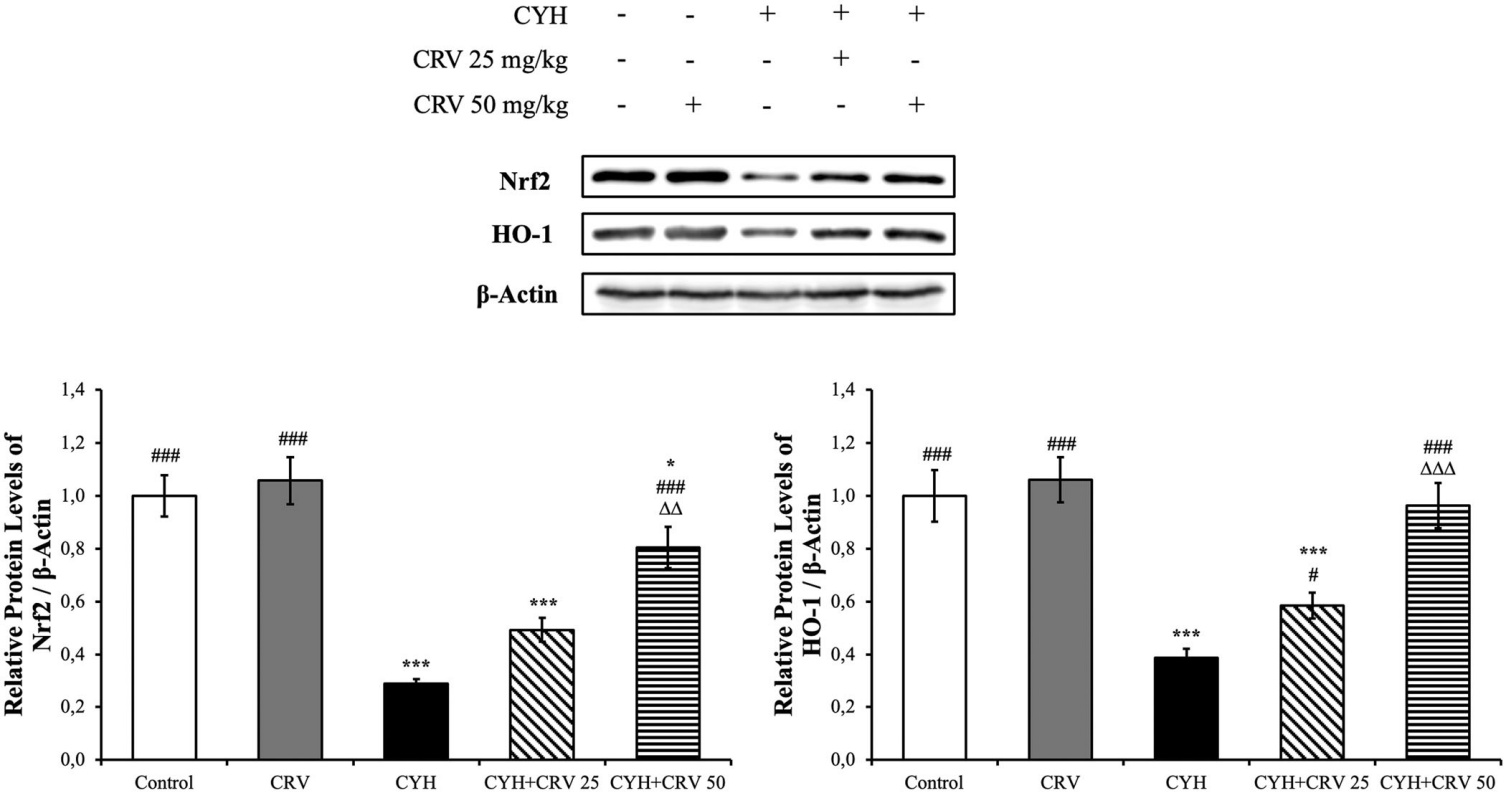
Effects of CYH and CRV administrations on Nrf2 and HO‐1 protein levels in sciatic nerve tissue of rats. Values are given as mean ± SD. Control vs others: ^⁎⁎⁎^ *p* < 0.001 and ^⁎^ *p* < 0.05. CYH vs Others; ^###^ *p* < 0.001, CYH + CRV 25 vs CYH + CRV 50: ^∆∆∆^ *p* < 0.001 and ^∆∆^ *p* < 0.01.

Nrf2 and HO‐1 protein levels were analyzed by Western blot (Figure 6). Nrf2 and HO‐1 levels were decreased in the CYH group compared to the control group (*p* < 0.001). On the other hand, CRV increased only HO‐1 levels at a dose of 25 mg/kg (*p* < 0.05), while it increased both Nrf2 and HO‐1 levels at a dose of 50 mg/kg (*p* < 0.001). The 50 mg/kg dose was more effective when CRV doses were compared (Nrf2: *p* < 0.01 and HO‐1: *p* < 0.001).

**Figure 7:**
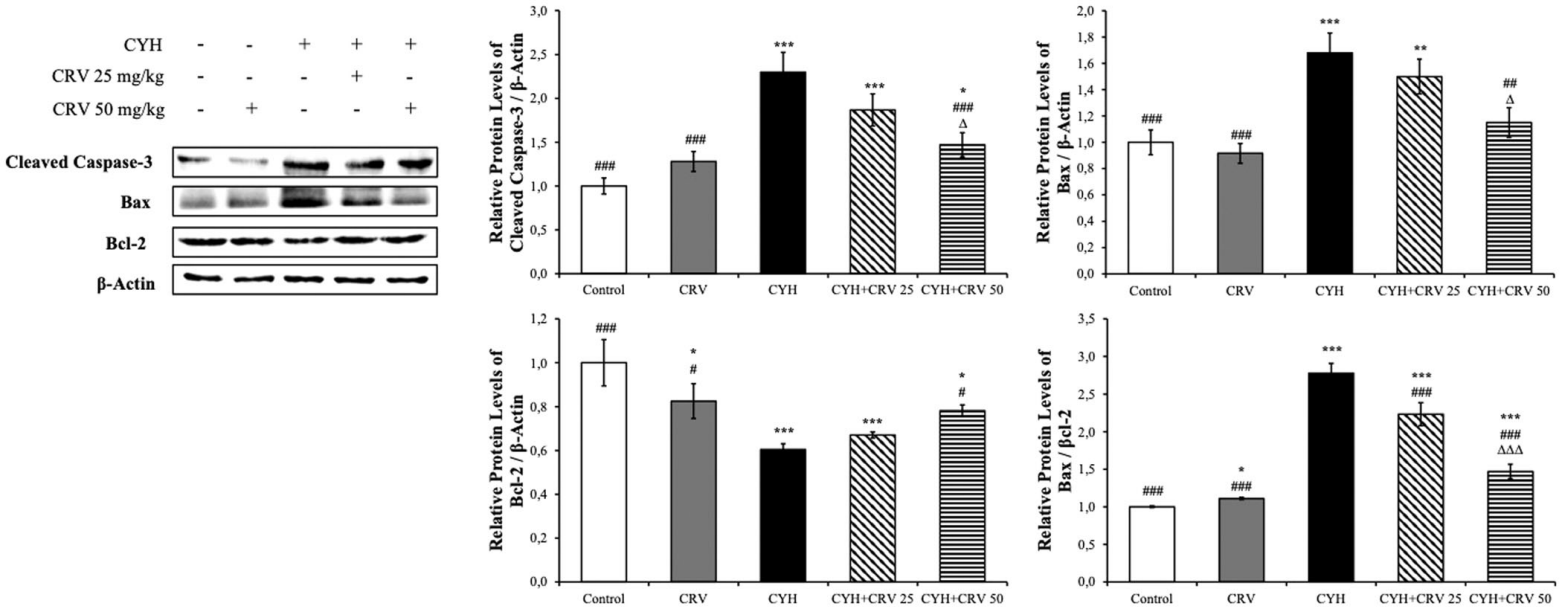
Effects of CYH and CRV administrations on Cleaved Caspase‐3, Bax, and Bcl‐2 protein levels in sciatic nerve tissue of rats. Values are given as mean ± SD. Control vs others: ^⁎⁎⁎^ *p* < 0.001, ^⁎⁎^ *p* < 0.01, ^⁎^ *p* < 0.05. CYH vs Others; ^###^ *p* < 0.001, ^##^ *p* < 0.01, ^#^ *p* < 0.05, CYH + CRV 25 vs CYH + CRV 50: ^∆∆∆^ *p* < 0.001 and ^∆^ *p* < 0.05.

Cleaved Caspase‐3, Bax, and Bcl‐2 protein levels were also analyzed by Western blot (Figure 7). In the CYH group, apoptotic Caspase‐3 and Bax levels increased compared to the control group, while antiapoptotic Bcl‐2 levels decreased. Moreover, the Bax/Bcl‐2 ratio increased. When CRV was administered with CYH, this situation was reversed compared to the CYH group. CRV was more effective at high doses (significance levels are detailed in Figure 7).

### 3.8 Results of Evaluation of Sciatic Nerves Using Immunohistochemical Methods

**Figure 8:**
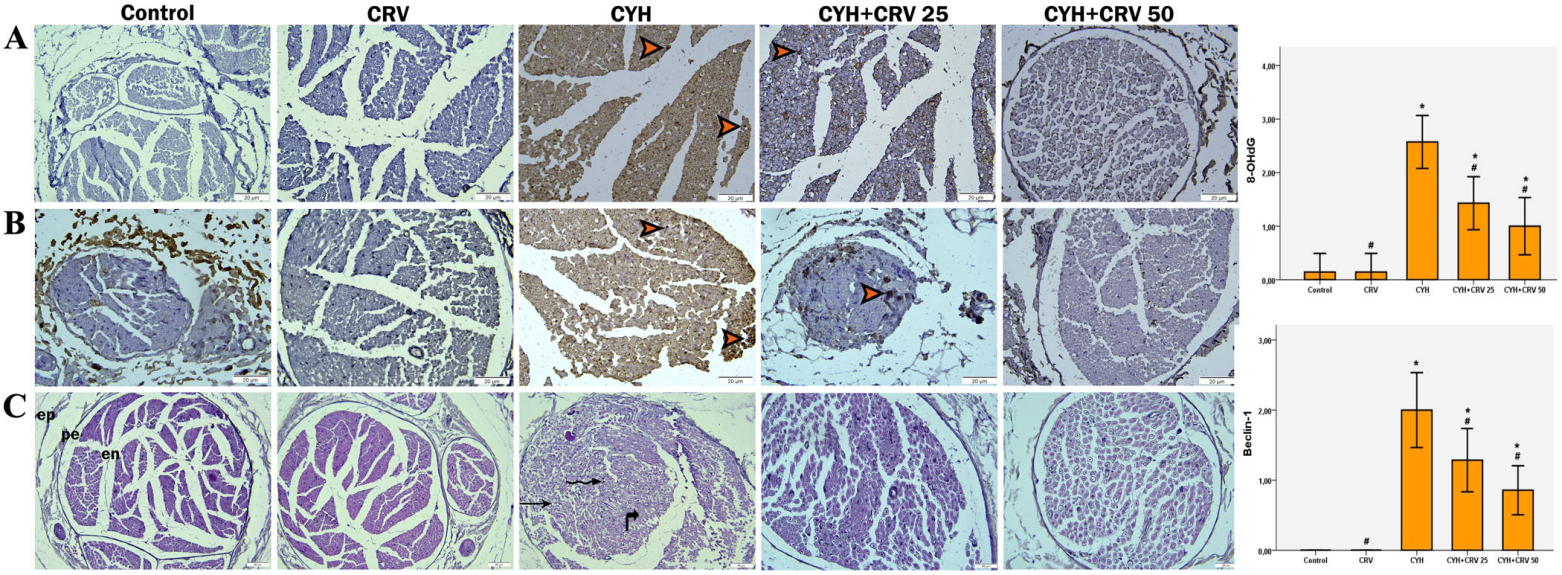
Immunohistochemistry and hematoxylin and eosin (H&E)‐stained photomicrograph of rat sciatic nerve sections of each group; In immunohistochemical staining (×40 magnification), photomicrographs of sagittal sections of the sciatic nerve of control and CRV‐treated rats showed minimal staining for 8‐OHdG (A) and beclin‐1 (B), in the CYH group, 8‐OHdG (A) and beclin‐1 (B) immunoreactivity showed high‐intensity staining (arrowheads), in the groups receiving CRV treatment (25 and 50 mg/kg), 8‐OHdG (A) and beclin‐1 (B) immunoreactivity showed moderate staining (arrowhead). In H&E staining (×20 magnification), Figure (C). Photomicrographs of sagittal sections of the sciatic nerve of control and CRV‐treated rats showed normal nerve fiber bundles, normal connective tissue sheaths (ep: epineurium, pe: perineurium, en: endoneurium), vacuolated axons (curved arrow), degenerated axon structures (curved arrow), axonless nerve fibers (thin arrows) in CYH group, CRV treatment (25 and 50 mg/kg) reduced the pathological changes induced by CYH, and an appearance close to control was obtained. Data are expressed as means ± SD from seven animals for each group. *p < 0.05 versus control group, ^#^ *p* < 0.05 versus CYH group.

The 8‐OHdG immunoreaction of the sciatic nerve sections in the control and CRV groups was observed to be very mild as seen in Figure 8A. When compared to the control, there was a significant increase in the 8‐OHdG immunoreaction in the CYH group. In the treatment groups that received CRV together with the CYH group, scattered and reduced 8‐OHdG immunoreaction was detected. When the micrographs of the control and CRV groups were examined, it was seen that the beclin‐1 immunoreaction was low in Figure 8B, while an increase was detected in the CYH group. In both CRV dose treatments, there was a significant decrease in the beclin‐1 immunoreaction compared to the CYH group. There was no statistically significant difference between the CYH + CRV 25 and CYH + CRV 50 doses.

### 3.9 Results of Morphological Evaluation of Sciatic Nerves

The morphology of sagittal sections of sciatic nerve tissue was evaluated according to H&E staining and photomicrographs are presented in Figure 8c. Sciatic nerve tissue in the control and CRV‐treated groups showed normal histological structure. The endoneurium, the connective tissue sheath surrounding each nerve fiber in the healthy structure, and the perineurium, the connective tissue sheath surrounding the nerve fascicle, were regular. On the other hand, loss of myelin sheaths, neuronal degeneration, swelling of axons, and vacuolization were seen in axons in the CYH‐treated group. In addition, the nerve fibers in this group were loosely packed and irregularly distributed. The damage induced by CYH was largely repaired in the CYH + CRV‐treated groups. While there was a decrease in vacuolation and degeneration, the organization of nerve fibers was more regular. As a result, the administration of CRV treatment at different doses showed morphology close to the control without causing any deterioration in nerve fibers according to H&E staining results.

## 4 Discussion

Lambda‐cyhalothrin is a widely used synthetic pyrethrin insecticide that affects many organs [18]. It is known that it accelerates neuroinflammation in addition to different organ and tissue damage, thus impairing the function of nerve cells [19]. In the presented study, the possible mechanisms of action of CRV were investigated in rats with CYH‐induced sciatic damage.

It is known that pyrethrins cause continuous neuronal discharge and as a result, neuropathy develops [1]. In the presented study, neuropathic pain and heat sensitivity behavioral tests were performed and it was determined that CYH administration increased the development of peripheral neuropathy. It was also found that the combined administration of CRV and CYH was effective in alleviating peripheral neuropathy, as evidenced by behavioral tests, and restored the measured parameters to near‐control levels.

Disruption of oxidant and antioxidant balance in tissues causes oxidative stress [20, 21]. It is known that CYH causes oxidative stress through ROS production and can change enzyme systems that scavenge antioxidants or free oxygen radicals, and the resulting ROS disrupts cell functions by affecting macromolecules such as lipids, proteins, and DNA [21]. Lipid peroxidation (LPO), as a cellular damage mechanism, can be used as an indicator of oxidative stress [22]. ROS react with phospholipids, enzymes, and membrane receptors to form products such as 4‐hydroxynonenal and MDA, thus changing the fluidity of the cell membrane and the function of the cell [23, 24]. The antioxidant property of GSH is because it breaks down the free radicals and keeps them in an oxidized state [25, 26]. Endogenous antioxidant enzymes are the first defense mechanism against oxidative stress caused by ROS in cells due to their ability to eliminate free radicals [27]. One of the markers widely used in the assessment of oxidative stress is the Nrf2/HO‐1 signaling pathway [28]. Nrf2 is a transcription factor that plays a role in regulating the balance of GSH and antioxidant enzyme levels [29, 30]. HO‐1 is one of the genes regulated by Nrf2 [31]. Nrf2 and Keap1 are found in a combined form in healthy cells. When any damage occurs with various stimuli, Nrf2 and Keap1 are separated and Nrf2 is transported to the nucleus. When it passes into the nucleus, HO‐1 activates cellular antioxidants such as NQO1, GCLM, and GCLC. GCLM and GCLC play a role in GSH synthesis [32]. In the presented study, it was determined that Lambda‐cyhalothrin caused damage to the cell membrane by increasing the level of MDA in sciatic tissue, and at the same time, it suppressed the expression level of enzymes responsible for the antioxidant defense system such as Nrf2, HO‐1, NQO1, GCLM, and GCLC, and caused the development of oxidative stress. Additionally, it was determined that CRV administration was effective in reducing CYH‐induced sciatic tissue oxidative stress, and achieved this by reducing lipid peroxidation and increasing antioxidant enzyme expressions. It has been shown in different studies that CYH causes tissue damage, increases MDA levels and induces oxidative stress by decreasing antioxidant enzyme activities [4, 13, 33, 34]. Similarly, Xu et al. [6] reported that exposure to CYH increases oxidative stress and disrupts cellular functions. It was revealed in the study conducted by Gencer et al. [35] that CRV is effective in reducing lipid peroxidation and strengthens the antioxidant system, especially by activating the Nrf2/HO‐1 pathway.

Inflammation is a cell defense that develops immediately in response to infection or tissue damage [36]. One of the most well‐known intracellular signaling pathways of inflammatory responses is the NF‐κB signaling pathway [37]. The induction of cytokine expression by environmental chemicals initiates the inflammatory response, and cytokines are considered important markers of the inflammatory response to environmental chemicals [38]. Pesticides cause increased secretion of inflammatory cytokines (TNF‐α, IL‐1β), thereby accelerating inflammation [39]. TNF‐α, a cytokine produced by activated macrophages, is an important mediator of local and systemic inflammation. IL‐1β, together with TNF‐α, plays an important role in the initiation of inflammatory processes by regulating the expression of other cytokines and chemokines [40]. It has been suggested that lambda‐cyhalothrin increases ROS production and that increased ROS production activates TNF‐α, IL‐1β and other genes that play a role in inflammation through the stimulation of NF‐κB, a transcription factor located at the transition point of inflammatory and oxidative stress [14, 41, 42]. Another factor that plays an important role in accelerating the inflammatory process is RAGE. It accelerates inflammation by activating the NF‐κB signaling pathway. It takes part in inflammasomes, which consist of a group of protein complexes, in the regulation of the inflammatory response. One of these proteins, NLRP3, is activated by exogenous stimuli and stimulates the maturation and release of pro‐inflammatory cytokines [43]. In the presented study, it was found that CYH administration increased NF‐κB, TNF‐α, IL‐1β, RAGE, and NLRP3 expressions in parallel with the increase in ROS production in sciatic tissue and accelerated the inflammation process, while CRV administration together with CYH was effective in suppressing inflammation by reversely regulating these cytokine levels. It is thought that CRV provides this effect, especially by reducing ROS production, that decreased ROS production is insufficient to stimulate inflammation, and that the cell, which is freed from the effects of ROS and inflammation, continues its normal functions. It has been reported that CRV, which is used due to its anti‐inflammatory effect in various toxicity models, particularly suppresses NF‐κB expression, and that suppressed NF‐κB cannot stimulate the expression of inflammatory markers, especially TNF‐α and IL‐1β, and is effective in reducing inflammation [44, 45, 46].

The ER is a vital cellular organelle with important functions such as the formation and modification of the three‐dimensional structure of proteins, biosynthesis of cell membrane proteins, calcium homeostasis, and lipid and steroid biosynthesis [47]. Environmental and chemical factors lead to Ca imbalance in the ER lumen and the accumulation of unfolded or misfolded proteins [48]. Unfolded proteins are detected by transmembrane sensors (IRE1, ATF‐6, PERK, CHOP) and cause the unfolded protein response (UPR) in cells to restore cellular homeostasis [49, 50]. As the presence of stress continues, the UPR continues to prolong and endoplasmic reticulum (ER) stress develops. Oxidative stress is one of the factors that trigger ER stress and IRE1, ATF‐6, PERK, and CHOP are important ER stress markers, ER stress is characterized by increases in the expression levels of these markers [51]. It has been revealed in different studies that pesticides and Lambda‐cyhalothrin increase the expressions of IRE1, ATF‐6, PERK, and CHOP and thus expose the cell to the effect of ER stress [13, 52, 53]. In the presented study, it was determined that CYH exposure induced ER stress by increasing the expression levels of IRE1, ATF‐6, PERK, and CHOP in the sciatic tissue, and CRV‐supportive treatment given together with CYH was effective in suppressing ER stress. Evyapan et al. [54] reported that CRV reduces the activation of IRE1, ATF‐6, PERK, and CHOP in rats and suppresses the formation of ER stress, thus being effective in protecting the cell.

The increase in ROS production weakens the antioxidant defense system and causes damage to DNA and protein structures. The developing damage induces cellular apoptosis [55]. Prolonged ER stress also supports the process [56]. Apoptosis eliminates damaged and dangerous cells [57], as well as damaging healthy cells, and p53, caspase‐3, ‐6, ‐9, Apaf‐1, Bax, and Bcl‐2, are the most important proteins of the apoptotic pathway that determines the line between life and death in the cell [58]. The apoptotic process begins with cytochrome c combining with Apaf‐1 and activating caspase‐9. Activated caspase‐9 also activates caspase‐3. Caspase‐3 is the most important member of the caspase family and determines the fate of the cell by competing with Bcl‐2 [59, 60, 61]. While Bax accelerates apoptosis by opening pores in the mitochondrial membrane, Bcl‐2 shows the opposite effect by preventing the opening of pores and shows an antiapoptotic effect. P53, on the other hand, prevents the proliferation of damaged DNA and cell growth and stimulates apoptosis to eliminate defective cells [62]. In the presented study, it was determined that CYH administration increased the expressions of p53, caspase‐3, ‐6, ‐9, Apaf‐1, and Bax in sciatic tissue, while decreasing the expression of Bcl‐2 and accelerating the apoptotic process. Increased ROS production and ER stress are thought to play a role in this process. Studies have shown that pesticides and Lambda‐cyhalothrin increase the expression of caspase‐3 and Bax in different organs, reduce Bcl‐2 levels, and thus accelerate the apoptotic process of the cell [13, 52, 53, 63]. It was determined that CRV administered together with CYH was effective at both doses, increased Bcl‐2 expression, and slowed down the apoptotic process by suppressing p53, caspase‐3, ‐6, ‐9, Apaf‐1 and Bax expressions, and showed antiapoptotic properties. Studies have shown that CRV reduces Bax and caspase‐3 levels in different toxicity models and different organs and is effective in protecting cells from apoptosis by increasing Bcl‐2 levels, and it has been stated that it would be beneficial to use it as a supportive treatment [35, 44, 45].

## 5 Conclusion

When the obtained data were evaluated together, it was determined that CYH accelerated inflammation by increasing oxidative stress in the sciatic tissue, and as a result of these, ER stress developed, the developing ER stress accelerated apoptosis and as a result, cell death increased, while CRV showed antioxidant, anti‐inflammatory and antiapoptotic effects and minimized the damage by reversing these processes in the cell. As a result, the effectiveness of CRV on CYH‐induced sciatic damage was seen and it was thought that it would be useful to use it as a supportive treatment.

## Author Contributions

**Özge Kandemir:** conceptualization, data curation, methodology, original draft preparation. **Mustafa İleritürk:** data curation, methodology. **Cihan Gur:** data curation, methodology. **Nurhan Akaras:** conceptualization, data curation, methodology. **Hasan Şimşek:** data curation, methodology. **Selçuk Yılmaz:** data curation, methodology. **Fatih Mehmet Kandemir:** conceptualization, data curation, methodology.

## Ethics Statement

The study was approved by Atatürk University Animal Experiments Local Ethics Committee (no. 2023/12‐178, date: 10/26/2023).

## Consent

The authors gave their explicit consent to publish the manuscript. Özge Kandemir is free to contact any of the people involved in the research to seek further clarification and information.

## Conflicts of Interest

The authors declare no conflicts of interest.

## Data Availability

Data will be made available on request.
